# DXO/Rai1 enzymes remove 5′-end FAD and dephospho-CoA caps on RNAs

**DOI:** 10.1093/nar/gkaa297

**Published:** 2020-05-06

**Authors:** Selom K Doamekpor, Ewa Grudzien-Nogalska, Agnieszka Mlynarska-Cieslak, Joanna Kowalska, Megerditch Kiledjian, Liang Tong

**Affiliations:** 1 Department of Biological Sciences, Columbia University, New York, NY 10027, USA; 2 Dept. Cell Biology and Neuroscience, Rutgers University, Piscataway, NJ 08854, USA; 3 Division of Biophysics, Institute of Experimental Physics, Faculty of Physics, University of Warsaw, 02-093 Warsaw, Poland

## Abstract

In eukaryotes, the DXO/Rai1 enzymes can eliminate most of the incomplete and non-canonical NAD caps through their decapping, deNADding and pyrophosphohydrolase activities. Here, we report that these enzymes can also remove FAD and dephospho-CoA (dpCoA) non-canonical caps from RNA, and we have named these activities deFADding and deCoAping. The crystal structures of mammalian DXO with 3′-FADP or CoA and fission yeast Rai1 with 3′-FADP provide elegant insight to these activities. FAD and CoA are accommodated in the DXO/Rai1 active site by adopting folded conformations. The flavin of FAD and the pantetheine group of CoA contact the same region at the bottom of the active site tunnel, which undergoes conformational changes to accommodate the different cap moieties. We have developed FAD-capQ to detect and quantify FAD-capped RNAs and determined that FAD caps are present on short RNAs (with less than ∼200 nucleotides) in human cells and that these RNAs are stabilized in the absence of DXO.

## INTRODUCTION

RNA modifications have emerged as key regulators of RNA function and metabolism. Recent advances in RNA sequencing techniques have uncovered novel RNA modifications and revealed their modes of regulation and impact on cellular processes and disease (1,2). RNA modifications at the 5′ end play a particularly significant role in the ‘epitranscriptomic’ pathway because the 5′ end regulates most aspects of the life cycle of RNA, from biogenesis to decay (3–8). In eukaryotes, the most common, or canonical, 5′-end modification of mRNAs is the N^7^-methylguanosine (m^7^G) cap, which is converted from the nascent 5′ triphosphate by three enzymatic reactions (3). The m^7^G cap is important for processing, stability, transport and translation (4). This cap can undergo further modifications including 2′-*O*-methylation of the first few nucleotides which enables cellular discrimination of self from non-self, and adenosine N^6^ methylation in the cap, which can promote RNA stability as well as enhance translation efficiency (5,9,10).

Recently, it was demonstrated that RNAs can also be modified at their 5′ end with the cofactor and metabolite NAD^+^ (6). The NAD^+^ cap was initially identified in bacteria and was demonstrated to be appended to RNA by employing NAD^+^ as a non-canonical initiating nucleotide during transcription (11–13). The NAD^+^ cap can protect the 5′ end of RNAs in bacteria from RNases requiring an unprotected 5′ end, such as RNase E, but can be hydrolyzed by NudC, a Nudix family protein, releasing nicotinamide mononucleotide (NMN) and 5′ monophosphate RNA (12,14). Eukaryotic NAD^+^-capped RNA was subsequently identified in yeast and human cells, and these RNAs could be spliced and polyadenylated (15,16). In mitochondria, NAD^+^-capped RNA levels can reach up to 60% of mitochondrial transcripts, which appear to be linked to cellular metabolism and can be influenced by environmental conditions (17–19). In contrast to bacterial NAD^+^ caps, eukaryotic NAD^+^ caps can promote RNA decay through the activity of the decapping enzyme DXO (20,21), a process called deNADding (15). The eukaryotic Nudix protein Nudt12 can hydrolyze NAD^+^ caps on distinct transcripts compared to DXO (19). Finally, NAD^+^-capped RNA has also been identified in plants and demonstrated to be enriched on translating ribosomes (22,23), and the plant homolog of DXO can catalyze removal of NAD^+^ from RNA (24).

The DXO/Rai1 family of proteins consists of mammalian DXO and fungal Rai1 and Dxo1. They hydrolyze incompletely-capped mRNAs in a quality control mechanism to maintain 5′-end integrity (25). DXO enzymes are capable of removing the entire cap structure by hydrolyzing the phosphodiester linkage between the first and second encoded nucleotides, releasing GpppN from unmethylated capped RNA (GpppN-RNA) (21,26,27), and they can release the intact NAD^+^ from NAD^+^-capped RNA (15). DXO, Dxo1 and some Rai1 homologs also possess 5′-3′ exonuclease activity and can both decap and degrade incompletely-capped or NAD^+^-capped RNAs (15,21,27,28). A single active site is used for all the DXO/Rai1 hydrolytic activities, which are mediated by binding of the scissile phosphate to two divalent cations (magnesium or manganese) that are coordinated by conserved acidic residues (21). A strictly conserved arginine residue recognizes the phosphate group(s) in the cap dinucleotide.

Aside from NAD^+^, RNA can also be modified by other non-canonical caps such as flavin adenine dinucleotide (FAD), dephospho-CoA (dpCoA) and UDP factors UDP-Glc and UDP-GlcNAc (8,13,17,29–32). *Schizosaccharomyces pombe* Rai1 (SpRai1) can remove dpCoA and FAD caps *in vitro* (33), but it remains unclear if this activity extends to other DXO/Rai1 homologs. Here we report that mammalian DXO, *Kluyveromyces lactis* Dxo1 (KlDxo1) and SpRai1 can remove FAD and dpCoA caps from RNA *in vitro*. The crystal structures of mouse DXO in complex with 3′-FADP and CoA reveal the molecular basis for the recognition of these diverse non-canonical caps by DXO. We also report the crystal structure of SpRai1 in complex with FAD and demonstrate how DXO/Rai1 homologs recognize the folded conformation of non-canonical caps by distinct mechanisms. We have developed the FAD-capQ method and used it to detect FAD-capped RNAs in human cells. We show that FAD-capped RNAs are present on short RNAs and upregulated in the absence of DXO, indicating a regulatory role for DXO in the control of FAD-capped RNAs in cells.

## MATERIALS AND METHODS

### Protein expression and purification

His-tagged full-length wild-type and mutant mouse DXO (mDXO) and full-length KlDxo1 in pET28a or full-length SpRai1 in pET28a (for crystallization) or pET26b (for assays) were used to transform *Escherichia coli* BL21 (DE3) Rosetta cells. DXO and SpRai1 mutant proteins were generated using the Quikchange site-directed mutagenesis kit (Stratagene). Proteins were expressed and purified as previously reported (20). Briefly, mDXO/SpRai1 expression was induced with 0.3 mM IPTG at 18°C overnight and purified with Ni-NTA Superflow (Qiagen) and gel filtration (Sephacryl S-300) chromatography (in running buffer containing 20 mM Tris (pH 7.5), 250 mM NaCl and 2 mM DTT). Proteins were supplemented with 5% (v/v) glycerol and concentrated to 10 mg/ml before being frozen in liquid nitrogen.

### Crystallization, data collection and structure determination

mDXO crystals were obtained using the hanging-drop vapor diffusion method at 20°C with a reservoir solution containing 21–26% (w/v) PEG 3350. For the 3′-FADP complex structure, mDXO crystals were soaked in 10 mM FADP (ammonium salt) in water for 1 h. For the CoA complex structure, crystals were soaked in 20 mM CaCl_2_ for 1 h followed by soaking in 10 mM CoA (sodium salt) in a new drop for additional 2 h at 20°C in buffer containing 25% (w/v) PEG 3350 and 0.1 M Tris (pH 6.8).

SpRai1 crystals were obtained using the hanging-drop vapor diffusion method at 20°C with a reservoir solution containing 10% (w/v) PEG 3350 and 0.2 M MgCl_2_. SpRai1 crystals were soaked with 10 mM FADP (ammonium salt) in water for 1 h. All the crystals were cryoprotected with 25% (w/v) PEG 3350 and 25% (v/v) ethylene glycol before being flash frozen in liquid nitrogen for diffraction analysis and data collection at 100 K.

X-ray diffraction data were collected at the Advanced Photon Source (APS) beamline 24-ID-E and the diffraction images were processed and scaled using the XDS program (34). The crystals are isomorphous to those reported earlier (20). The structure refinement was initiated with a rigid-body refinement in Phenix (35) with mDXO (PDB entry 4J7L) or SpRai1 (PDB entry 3FQG) as the initial model. Further structure refinement was performed using PHENIX and the atomic model was built with the Coot program (36). No electron density was observed for residues at the N (1–26, together with the His tag) and C (385–397) termini of mDXO, and they are not included in the atomic model. The crystallographic information is summarized in Table 1.

**Table 1.** Summary of crystallographic information

**Structure**	mDXO mutant + 3′-FADP	mDXO mutant + CoA	SpRai1 mutant + 3′-FADP	SpRai1 wild-type + 3′-FADP
**Data Collection**				
Space group	*P*2_1_	*P*2_1_	*C*2	*C*2
Cell dimensions				
*a*, *b*, *c* (Å)	50.0, 87.9, 53.3	49.9, 87.7, 54.0	101.3, 60.4, 72.8	100.6, 60.0, 72.5
α, β, γ (º)	90, 113.4, 90	90, 112.2, 90	90, 104.1, 90	90, 104.7, 90
Resolution (Å)^a^	48.9–1.6 (1.66–1.6)	46.2–1.6 (1.66–1.6)	45.9–1.9 (1.97–1.9)	48.7–1.9 (1.97–1.9)
*R*_merge_ (%)	4.1 (31.8)	2.8 (46.7)	2.0 (58.0)	3.3 (51.7)
*I*/σ*I*	14.3 (2.6)	14.0 (1.5)	18.6 (1.9)	16.8 (1.9)
Completeness (%)	99.8 (97.5)	99.6 (98.2)	99.1 (98.6)	98.3 (97.9)
No. of reflections	54743	56497	33335	32425
Redundancy	1.9 (1.9)	2.0 (2.0)	2.0 (2.0)	1.9 (1.9)
**Refinement**				
Resolution (Å)	48.9–1.6 (1.66–1.6)	49.9–1.6 (1.66–1.6)	45.9–1.9 (1.97–1.9)	48.7–1.9 (1.97–1.9)
*R*_work_ (%)	19.9 (27.7)	18.5 (42.5)	17.2 (26.8)	19.1 (29.4)
*R*_free_ (%)	23.4 (28.6)	21.4 (44.0)	21.0 (26.9)	22.9 (31.0)
Number of atoms	3343	3349	2777	2800
Protein	2910	2921	2581	2566
Ligand/Ion	61	48	57	64
Water	372	380	139	170
*B*-factors (Å^2^)	25.8	29.3	52.9	45.8
Protein	24.1	28.2	52.9	45.6
Ligand	30.2	31.7	49.1	41.0
Water	38.5	38.2	55.5	50.4
r.m.s.d.				
Bond lengths (Å)	0.010	0.012	0.010	0.011
Bond angles (°)	1.33	1.17	1.33	1.40

^a^The numbers in parentheses are for the highest resolution shell.

### Cell culture and RNA extraction

Human embryonic kidney HEK293T cells were obtained from ATCC. Cells were cultured in DMEM medium (Thermo Fisher Scientific) supplemented with 10% fetal bovine serum (Atlanta Biologics), and antibiotics (100 units/ml penicillin and 100 μg/ml of streptomycin) under 5% CO_2_ at 37°C. The three different monoclonal HEK293T cell lines harboring CRISPR-Cas9n double-nick generated distinct homozygous deletions within the *DXO* gene genomic region corresponding to its catalytic site have previously been reported (15). Total cellular RNAs were extracted with TRIzol Reagent according to the manufacture's protocol (Thermo Fisher Scientific).

### *In vitro* transcription of NAD, FAD, dpCoA-capped RNAs

**Figure 1.**
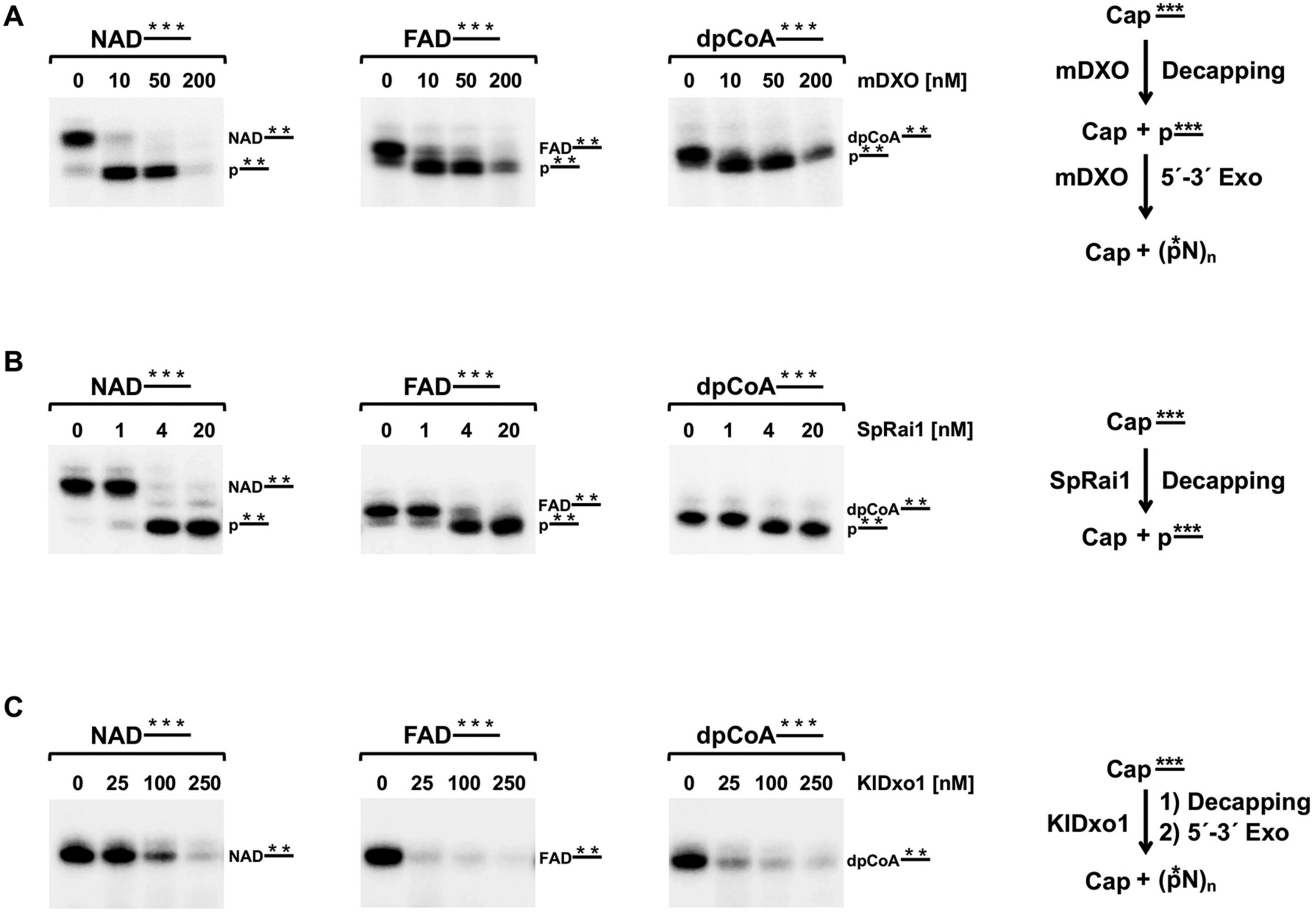
The DXO family of proteins cleave RNA capped with nucleotide metabolites. Schematics of NAD, FAD, or dpCoA-capped RNA hydrolysis by the DXO family enzymes are shown on the right. ^32^P-uniformly labeled RNAs (100 nM) capped with NAD, FAD or dpCoA as indicated were subjected to hydrolysis with mDXO (**A**), SpRai1 (**B**) or KlDxo1 (**C**) proteins *in vitro*. Data are derived from at least three independent experiments. The respective deNADding, deFADding and deCoAping products were resolved on 20% urea polyacrylamide gels without (C) or with 0.2% APB (A, B).

For assays shown in Figure 1, ^32^P-uniformly-labeled RNAs capped with either NAD, FAD or dpCoA were synthesized using ssDNA oligo ϕ2.5AG-30 nt as described (37). For assays shown in Figure 5B, FAD-capped RNA was generated by in vitro transcription of PCR generated ϕ2.5A-CA2 linear template (15). Both templates contain the T7 ϕ2.5 promoter that retained the first adenosine at the transcription start site and replaced all other adenosine with uracils. *In vitro* transcription was carried out at 37°C for 90 min in 50 μl of the reaction mixture containing 400 ng PCR-generated DNA template, 10 μl of 5 × transcription buffer, 5 μl of 10 mM DTT, 5 μl of 10 μg/μl BSA, 5 μl of 5 mM rCTP, 5 μl of 5 mM rUTP, 20 μCi [α-^32^P]GTP, 2 μl of T7 RNA polymerase (Promega), and 1 μμl of 40 U/μl RNasin Ribonuclease Inhibitor (Promega). Reactions were incubated for 90 min at 37°C and then treated with 3 units of DNase RQ1 (Promega) for 20 min at 37°C. Reactions were passed through G-50 columns (GE Healthcare) to remove unincorporated nucleotides, followed by extraction with acidic phenol:chloroform and RNA recovered by ethanol precipitation.

### RNA *in vitro* decapping assays

^32^P-labeled RNAs capped with either NAD, FAD or dpCoA were incubated with the indicated amount of recombinant proteins in decapping buffer containing 10 mM Tris (pH 7.5), 100 mM KCl, 2 mM DTT, 2 mM MgCl_2_ and 2 mM MnCl_2_ as previously described (26) and incubated at 37°C for 30 min. Reactions were stopped by the addition of RNA loading buffer (95% (v/v) deionized formamide, 30 mM EDTA, xylene cyanol, bromophenol blue), and decapping products were resolved using denaturing 20% (w/v) polyacrylamide gel containing 0.2% (w/v) acryloylaminophenylboronic acid. 0.5× TBE was used as gel running buffer. Reaction products were visualized with an Amersham Typhoon Biomolecular Imager (Ge Healthcare).

### FAD-cap detection and quantitation (FAD-capQ)

To minimize any copurifying free FAD in the RNA preparation that may lead to aberrant positive signal in the FAD-capQ assay, RNA initially derived by TRIzol Reagent purification (Thermo Fisher Scientific) was further purified using RNeasy columns (Qiagen). To examine short and long RNA fractions, total RNA (500 μg) was subjected to LiCl precipitation (2 M final concentration). After 30 min incubation at −80°C, samples were centrifuged at 16 000 × g at 4°C for 30 min to precipitate RNAs larger than ∼200 nucleotides and the resulting pellet was washed with 70% (v/v) ethanol. The short RNA population within the supernatant of the original LiCl precipitation was collected and appropriate amount of RLT buffer was added according to the Qiagen protocol. Two volumes of 100% ethanol were subsequently added to the RNA-RLT buffer mixture and samples were applied to RNeasy columns according to the manufacturer (Qiagen) and purified RNA eluted with water. To remove trace amounts of LiCl, 50 μg of obtained RNA was further purified using RNeasy column according to Qiagen protocol.

3 μg of short or long RNA fractions were subjected to FAD-cap Detection and Quantitation (FAD-capQ) assay. In the first step, RNA was digested with 200 ng of SpRai1 in 15 μl reaction containing 50 mM Tris (pH 7.9), 100 mM NaCl, 10 mM MgCl_2_ and 1 mM DTT at 37°C for 2 h to release intact FAD. The control samples were prepared by incubating 3 μg of RNA under the same reaction conditions with 200 ng of SpRai1 catalytically inactive double-mutant E199A/D201A. FAD standard curve was prepared under the same reaction conditions using increasing amount of FAD-capped RNA. Three micrograms of total RNA was added after the reaction to correct for background fluorescence. After a 2 h incubation at 37°C, 35 μl of FAD Assay Buffer (FAD Colorimetric/Fluorometric Assay Kit, BioVision) was added to each sample. In the second step, 50 μl samples were used in the fluorometric assay to measure OxiRed probe fluorescence (Ex/Em = 535/587 nm) according to the manufacture's protocol (FAD Colorimetric/Fluorometric Assay Kit, BioVision). The samples were incubated overnight in a 96-well plate at room temperature protected from light. The fluorescence signal was measured using Amersham Typhoon Biomolecular Imager (GE Healthcare) (Ex/Em = 532/570 nm). The signal corrected for the background fluorescence is proportional to concentrations of FAD within a sample.

FAD-capQ has two unique features compared with previously described NAD-capQ assay. First, SpRai1 was used in the FAD-capQ assay instead of Nuclease P1 which was observed to interfere with the OxiRed probe fluorescence intensity over the time of the reaction. Second, lower amount of RNA was used in the FAD-capQ assay since higher quantities of RNA appeared to quench the probe signal, which may account for the lack of detection in the 50 μg total RNA sample.

### Synthesis of 3′-FADP

#### General information

RNAse T2 was purchased from MoBiTec (Germany). Riboflavin 5′- monophosphate sodium salt hydrate was purchased from Sigma Aldrich. The sodium salt was converted into triethylammonium salt by passing its aqueous solution through Dowex 50 W x 8 cationite in the TEA^+^ form. The collected eluate was evaporated under reduced pressure with addition of ethanol and dried over P_2_O_5_ to yield the triethylammonium salt as a yellow solid (sensitive to light).

Synthetic nucleotides were purified by ion-exchange chromatography on a DEAE Sephadex A-25 (HCO_3_^-^ form) column. Each reaction mixture was loaded onto the column and washed thoroughly with water to remove solvents and non-binding reagents (until no precipitation with AgNO_3_ solution). The products were eluted with triethylammonium bicarbonate buffer. Fractions containing the desired product were combined after UV-VIS and HPLC analysis.

The final synthetic product, FAD 2′,3′-cyclophosphate (2**′**,3′-cFADP), was additionally purified by semi-preparative RP HPLC using a Discovery RP Amide C-16 HPLC column (25 cm × 21.2 mm, 5 μm, flow rate 5.0 ml min^–1^) with UV detection.

Analytical RP HPLC was performed using SUPELCO Supelcosil LC-18-T HPLC column (4.6 × 250 mm, 5 μm, flow rate 1.3 ml min^–1^) with a linear gradient of 50% (v/v) methanol in ammonium acetate buffer (pH 5.9, 0.05 M) in 15 min and UV-detection at 254 nm. The structure of 2**′**,3′-cFADP was confirmed by ^1^H and ^31^P NMR spectroscopy and low-resolution mass spectrometry (ESI–). The structure of cPAP-Im was confirmed by RP HPLC and low-resolution MS.

Mass spectra were recorded on AB Sciex API 3200 spectrometer. NMR spectra were recorded at 25°C with a VARIAN 400 spectrometer at 400 MHz (^1^H NMR) and 162 MHz (^31^P NMR). ^1^H NMR chemical shifts were calibrated to sodium 3-trimethylsilyl-[2,2,3,3-D4]propionate (TSP) in D_2_O and for ^31^P NMR to H_3_PO_4_ (20%, v/v) in D_2_O as an external standard. ^1^H NMR signal assignments and identification were supported by COSY spectra analysis. The raw NMR spectroscopic data were processed using MestReNova v 12.0.2-20910 Software.

#### 2′,3′-Cyclophosphoadenosine 5′-phosphorimidazolide, (2**′**,3′-cPAP-Im)

Adenosine (997 mg, 3.73 mmol) was suspended in PO(OCH_3_)_3_ (20 ml) and stirred at 0°C for 5 min. POCl_3_ was added (1.71 g, 11.2 mmol, 3 equiv.) followed by addition of the second portion (3.43 g, 22.4 mmol, 6 equiv.) after 1 h. The reaction was allowed to slowly reach room temperature under continuous stirring. The reaction progress was analyzed by RP HPLC. After 24 h, the reaction mixture was poured into cold diethyl ether. The precipitate was centrifuged and re-dissolved in PO(OCH_3_)_3_ (20 ml) followed by addition of imidazole (1.37 g, 201 mmol, 54 equiv.). The reaction was quenched after 1 h by addition of 10 volumes of water. The product was isolated by DEAE Sephadex. The fractions containing the desired product were poured together and stored at −20°C. The yield (1.17 mmol, 31%) was determined by UV-VIS measurements (ϵ_260_ = 15020 ml/mmol/cm).

#### 2′,3′-cyclophospho FAD, 2**′**,3′-cFADP

A solution of *2****′****,3′-*cPAP-Im (87 mg, 0.14 mmol, 1 equiv.) in TEAB buffer was evaporated under reduced pressure in the presence of 2 ml of DMF with repeated additions of 96% (v/v) ethanol until DMF solution of *2****′****,3′-*cPAP-Im was obtained. To the solution were added riboflavin 5′-monophosphate triethylammonium salt (73 mg, 0.13 mmol, 1 equiv.) and zinc chloride (155 mg, 1.14 mmol, 8 equiv.). The reaction progress was analyzed by RP HPLC. After complete substrate consumption, the reaction was quenched by addition of 10 volumes of an aqueous solution of EDTA and NaHCO_3_ (10 and 5 g/l, respectively). After purification by ion-exchange chromatography the isolated product was contaminated with 2′3′-cyclophosphoadenosine 5′-phosphate, which has the same negative net charge. After additional purification by semi-preparative HPLC 2′,3′-cFADP was isolated as an ammonium salt (53 mg, 235 mOD, 0.021 mmol, 16%). The UV-VIS measurements were performed in 0.1 M phosphate buffer at pH 7.0 (ϵ_450_ = 113 00 ml/mmol/cm).

^1^H NMR (400 MHz, D_2_O, 25°C): *δ* 8.40 (s, 1 H, H_A_), 7.84 (s, 1 H, H_A_), 7.75 (s, 1 H, H_ArF_), 7.75 (s, 1 H, H_ArF_), 5.86 (d, *J*_H,H_ 6.8 Hz, 1 H, H_A2′_), 5.02–4.93 (m, 1 H), 4.74–4.67 (m, 2 H), 4.59–4.53 (m, 2 H), 4.41–4.28 (m, 4 H), 4.14–4.02 (m, 3 H), 3.92–3.87 (m, 1 H), 2.46 (s, 3 H, H_CH3F_), 2.40 (s, 3 H, H_CH3F_) ppm.

^31^P NMR (162 MHz, D_2_O, 25°C): *δ* 3.69 (s, 1 P, P_A3′_), -9.65 (d, *J*_P,P_ 21.0 Hz, 1 P), –10.30 (d, *J*_P,P_ 21.0 Hz, 1 P) ppm.

#### FAD 3′-phosphate, 3′-FADP

The conversion of 2**′**,3′-cFADP into 3′-FADP was achieved by enzymatic cleavage with RNAse T2. 2**′**,3′-cFADP was dissolved at 10 mg/ml concentration in 50 mM ammonium acetate buffer pH 7. RNAse T2 (10 U/1 mg of compound) was added and the solution was gently shaken (300 rpm) for 24 h at 37°C. The completeness of enzymatic cleavage was confirmed by HPLC and MS analyses. The solution was filtered through Amicon^®^ Ultra-4 10K Centrifugal Filter. The protein concentrate was discarded and flow-through fraction was collected and repeatedly freeze-dried to a constant mass affording 3′-FADP with recovery of ∼70%.

## RESULTS

### The DXO family of proteins cleave RNA capped with nucleotide metabolites *in vitro*

Considering the broad substrate utilization of incompletely-capped and NAD^+^-capped RNAs by DXO/Rai1 homologs, we asked whether these enzymes can also recognize and hydrolyze RNAs capped with other nucleotide metabolites, such as FAD and dpCoA. To test the susceptibility of these RNAs to the DXO family of enzymes, *in vitro* transcribed ^32^P uniformly labeled 30-nt long RNAs capped with either NAD, FAD or dpCoA were subjected to cleavage with mouse DXO (mDXO), KlDxo1 or SpRai1. The resulting products were resolved using denaturing polyacrylamide gel electrophoresis (PAGE). To follow the hydrolysis activities of mDXO and SpRai1, acryloylaminophenylboronic acid (APB) was added to the denaturing polyacrylamide gel to enhance separation of NAD-, FAD- or dpCoA-capped RNAs from their corresponding decapped 5′-monophosphate RNAs by electrophoretic mobility. The separation is based on the specific interaction of APB with the capped RNAs that impedes their migration but not migration of the uncapped RNA (38). As expected, NAD-capped RNA was efficiently decapped (deNADded) by mDXO to RNA 5′-monophosphate and further degraded by its 5′-3′ exonuclease activity, which was particularly well manifested at the highest enzyme concentration (Figure 1A) (15). Importantly, both FAD and dpCoA-capped RNAs were susceptible to mDXO cleavage (deFADding and deCoAping, respectively), analogous to that observed with NAD-capped RNA (Figure 1A). A similar decapping outcome was observed with SpRai1 where all three nucleotide metabolite caps were also hydrolyzed. However, consistent with the lack of intrinsic exonuclease activity for SpRai1 (20), the resulting decapped RNAs were not further degraded (Figure 1B).

Analysis of the decapping activities of KlDxo1 revealed that it too possessed deFADding and deCoAping activities (Figure 1C), but with one major distinction. Unlike mDXO where a decapped monophosphorylated intermediate was evident prior to subsequent degradation of the RNA (Figure 1A), a similar intermediate was not observed with KlDxo1. In fact, a processive decapping-exonuclease activity was observed for KlDxo1 with all three nucleotide metabolite caps tested (Figure 1C), suggesting minimal product inhibition for the second exonucleolytic activity following the initial decapping step. Collectively, our data reveal that mammalian and fungal DXO/Rai1 family enzymes have robust activity to cleave RNAs capped with FAD and dpCoA.

### Structural basis for recognition of 3′-FADP by mammalian DXO

**Figure 2.**
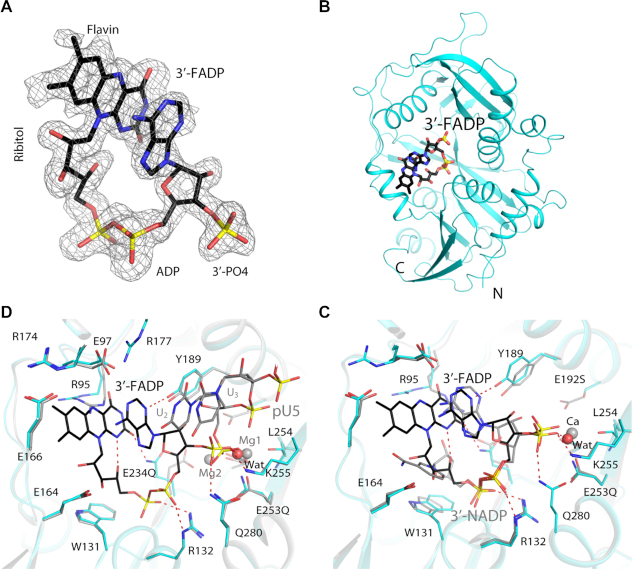
Crystal structure of mouse DXO in complex with 3′-FADP. (**A**) Simulated annealing omit *F*_o_– *F*_c_ electron density at 1.6 Å resolution for 3′-FADP, contoured at 2.5σ. (**B**) Overall structure of mouse DXO E192S/E234Q/E253Q mutant (cyan) in complex with 3′-FADP (in black for carbon atoms). (**C**) Overlay of the active site region of mouse DXO in complex with 3′-FADP (in color) with that in complex with 3′-NADP (gray; PDB: 5ULI) (15). The calcium ion from the 3′-NADP structure is shown in orange. (**D**) Overlay of the active site region of mouse DXO in complex with 3′-FADP (in color) with that in complex with pU5 RNA (gray; PDB: 4J7L) (21). The magnesium ions from the pU5 structure are shown in gray.

To elucidate the molecular mechanism for how the bulkier FAD (compared to NAD) is recognized by DXO, we attempted to determine the crystal structure of its complex with mouse DXO. Crystals of wild-type mDXO soaked with FAD with a 3′ phosphate (3′-FADP, hereafter called FADP) and catalytic divalent cations (magnesium or manganese) or cations which do not support regular activity (calcium) generated structures with electron density for only the diphosphate moiety of FADP. FADP was used so that its 3′ phosphate group could coordinate the metal ion(s) in the active site and provide additional interactions to the enzyme, similar to our earlier experiments with 3′-NADP (15). We then produced and crystalized several DXO mutants that we predicted would have no hydrolytic activity but would retain binding to FADP. These mutations were designed based on the proposed mechanism of hydrolysis (21) and the amino acid sequences of non-catalytic homologs of DXO, eIF3d and Cutoff (39,40). A structure with strong electron density for FADP (but not FAD) was finally obtained, at 1.6 Å resolution, by soaking the compound into crystals of the E192S/E234Q/E253Q triple mutant of mDXO (Figure 2A). Glu234 and Glu253 are direct ligands to the metal ions in the active site, while Glu192 is hydrogen-bonded to a water molecule on each metal ion (21). The refined atomic model has excellent agreement with the X-ray diffraction data and the expected bond lengths, bond angles and other geometric parameters (Table 1).

FADP is situated in the active site of DXO (Figure 2B) and is in a folded conformation, such that its adenine base and flavin are π-stacked to each other, at a distance of ∼3.5 Å (Figure 2C). The N3 atom of adenine is hydrogen-bonded to the side-chain hydroxyl of Tyr189, and the N1 atom is close to the side chain of Glu97, which is in an ion pair with Arg177. The N6 atom of adenine, which can be methylated in the m^7^G cap, is not involved in any direct contacts with mDXO. The carbonyl oxygen on C4 of flavin is hydrogen-bonded to the side chain of Arg95 and that on C2 to the side chain of E234Q. The N1 atom of flavin is hydrogen-bonded to a hydroxyl of the ribitol group, which is situated above and contacts the generally conserved Trp131 side chain. The flavin has a few direct contacts with the enzyme (for example the side chains of Glu97, Arg174 and Glu166), as well as interactions mediated through waters, for example Glu164 (Figure 2C).

The pyrophosphate of FADP interacts with the conserved Arg132 and Gln280 residues, and the distal phosphate relative to adenine is also hydrogen-bonded to the main-chain amides of Gly133 (at the N-terminal end of helix αB) and E234Q (a ligand to the second metal ion in wild-type DXO) (Figure 2C). The 3′ phosphate group interacts with the conserved Lys255 and Gln280 residues, as well as a water molecule occupying the position of the first metal ion. No metal ions were observed in the active site of this structure.

The binding mode of the pyrophosphate and the 3′ phosphate group of FADP is similar to that of 3′-NADP (15) (Figure 2C). On the other hand, the ribose and adenine have some differences in their orientation relative to the 3′ phosphate, and the difference between the nicotinamide ring and flavin positions is more pronounced. The nicotinamide and adenine rings of 3′-NADP are not π-stacked against each other, and Arg95 in that complex would clash with the flavin. A notable difference also exists for the side chain of Tyr189, to maintain hydrogen-bonding to the N3 atom of adenine, which is closer to the DXO surface. Besides these two residues, the other residues in the active site assume similar conformations in the two complexes.

A comparison to the binding mode of pU5 (penta-uridylate oligo RNA with a 5′-monophosphate) and Mg^2+^ ions (21) suggests the catalytic mechanism of the deFADding reaction and how DXO binds 5′-FAD RNA (Figure 2D). A rotation of −80° would bring the 3′ phosphate group of FADP to a similar position as that of the 5′ phosphate of pU5, where it can coordinate to the two metal ions in the active site and be correctly positioned for catalysis. The U1 base of pU5 stacks against the FADP adenine, and the flavin-adenine packing continues the base stacking pattern of the RNA body (Figure 2D). In this way, flavin mononucleotide of FAD mimics an additional nucleotide at the 5′ end of RNA.

### Structural basis for recognition of 3′-FADP by SpRai1

**Figure 3.**
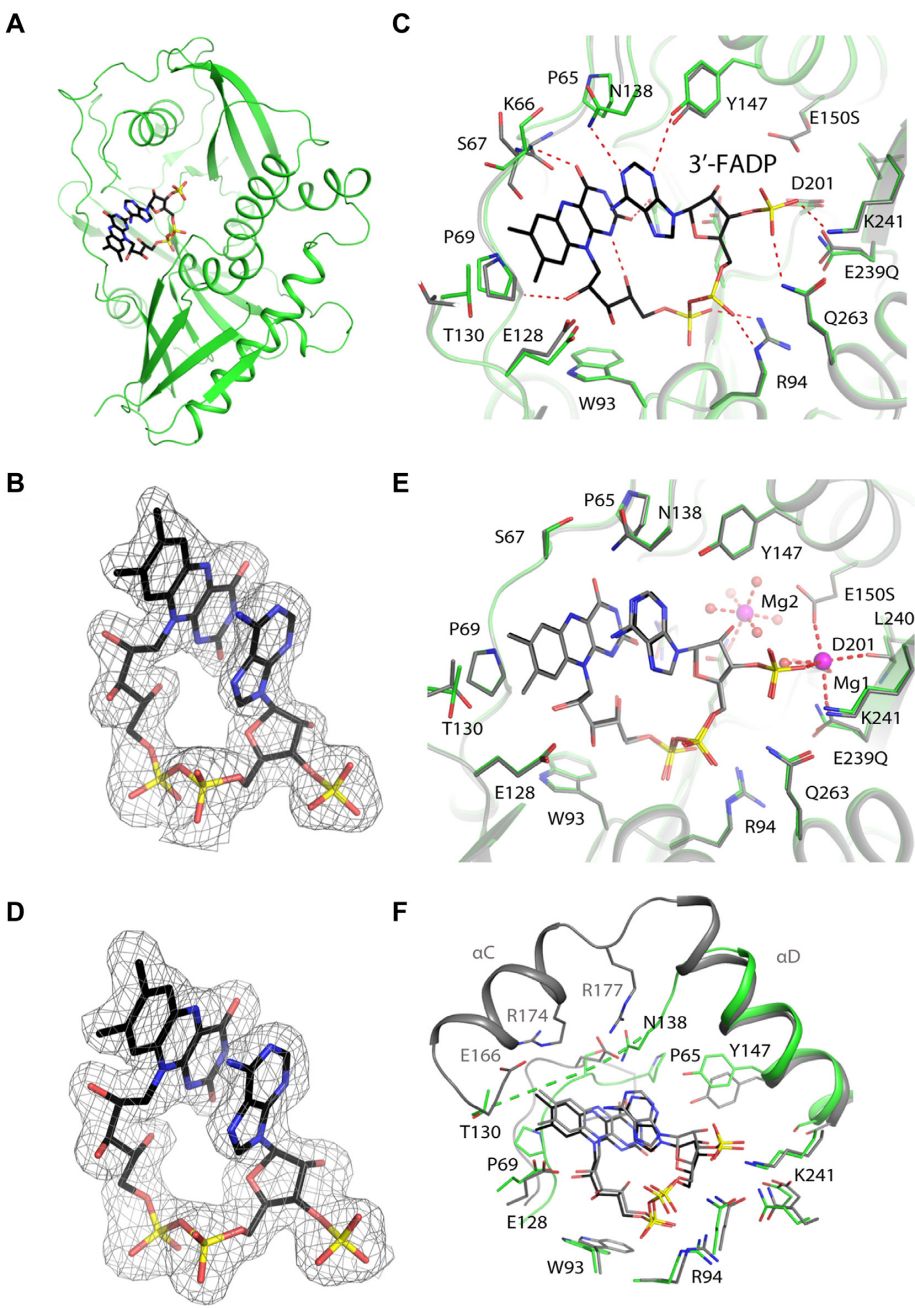
Crystal structures of SpRai1 in complex with 3′-FADP. (**A**) Overall structure of SpRai1 E150S/E199Q/E239Q mutant (green) in complex with 3′-FADP (in black for carbon atoms). (**B**) Simulated annealing omit *F*_o_– *F*_c_ electron density at 1.9 Å resolution for 3′-FADP in the complex with mutant SpRai1, contoured at 2.5σ. (**C**) Overlay of the active site region of SpRai1 in complex with 3′-FADP (in color) with that of free SpRai1 (gray; PDB: 3FQG) (20). (**D**) Simulated annealing omit *F*_o_– *F*_c_ electron density at 1.9 Å resolution for 3′-FADP in the complex with wild-type SpRai1, contoured at 2.5σ. (**E**) Overlay of the structure of mutant SpRai1 in complex with 3′-FADP (in color) with that of wild-type SpRai1 in complex with 3′-FADP (gray). The magnesium ions from the wild-type SpRai1 structure are shown in pink. (**F**) Overlay of the active site region of SpRai1 in complex with 3′-FADP (in color) with that of mDXO in complex with 3′-FADP (gray).

Modeling of FADP into the active site of free SpRai1 based on the mDXO complex indicated that FADP would clash with residues 65–71 (β4-αA loop) of SpRai1. Additionally, helix αC in DXO (containing residues 167–177) contributes to contacts with flavin (Figure 2C), but the equivalent residues are not ordered in the free SpRai1 structure. Therefore, to understand how SpRai1 functions as a deFADding enzyme, we determined the crystal structure of the equivalent triple mutant of SpRai1, E150S/E199Q/E239Q, in complex with FADP at 1.9 Å resolution (Figure 3A, Table 1).

Good-quality electron density was observed for FADP in the SpRai1 complex (Figure 3B), and it is also in a folded conformation. The structure shows that the backbone amide bonds for residues Lys66 and Ser67 flip by 180° to avoid the steric clashes between their carbonyls and flavin, which leads to a small conformational change for the β4-αA loop (Figure 3C). This conformational change also enables a hydrogen bond to be formed between the Ser67 backbone amide group and the C4 carbonyl oxygen of flavin. Pro65 and Pro69 in this loop have hydrophobic interactions with the flavin. The flavin and the ribitol groups also make other direct and water-mediated interactions with SpRai1, including Trp93 and Glu128 which are conserved in DXO (Trp131 and Glu164). Residues 131–137, equivalent to helix αC of mDXO, are still disordered in the FADP complex.

We were also able to observe good-quality electron density for FADP in complex with wild-type SpRai1 (Figure 3D), in a structure at 1.9 Å resolution (Table 1). The overall structure of the wild-type SpRai1 is essentially the same as that of the triple mutant, with rms distance of 0.2 Å for their equivalent Cα atoms, and the binding mode of FADP is essentially the same as well (Figure 3E), confirming that the mutations do not perturb the binding mode of FADP. Two magnesium ions were observed in the structure of the wild-type complex, one occupying metal binding site 1 and close to the 3′ phosphate of FADP. The other magnesium ion is coordinated by Glu199 and five waters, occupying a position that is similar to that seen for the third metal ion in the structure of *Candida albicans* Rai1 in complex with pU5 and manganese (28). The catalytic role of this metal ion (if any) is not known.

The bound conformation and position of FADP in SpRai1 are highly similar to those in mDXO (Figure 3F), indicating that the two enzymes have a conserved mechanism of recognizing this cap and catalyzing the deFADding reaction. Conserved residues between the enzymes generally maintain equivalent interactions with FADP, particularly the ADP moiety. Additionally, the ribitol group is contacted by a bulky tryptophan residue and glutamate residues. However, loop 65–71 of SpRai1, which contains residues that are not conserved in DXO, makes more intimate contacts to flavin.

### Structural basis for recognition of coenzyme A by DXO

**Figure 4.**
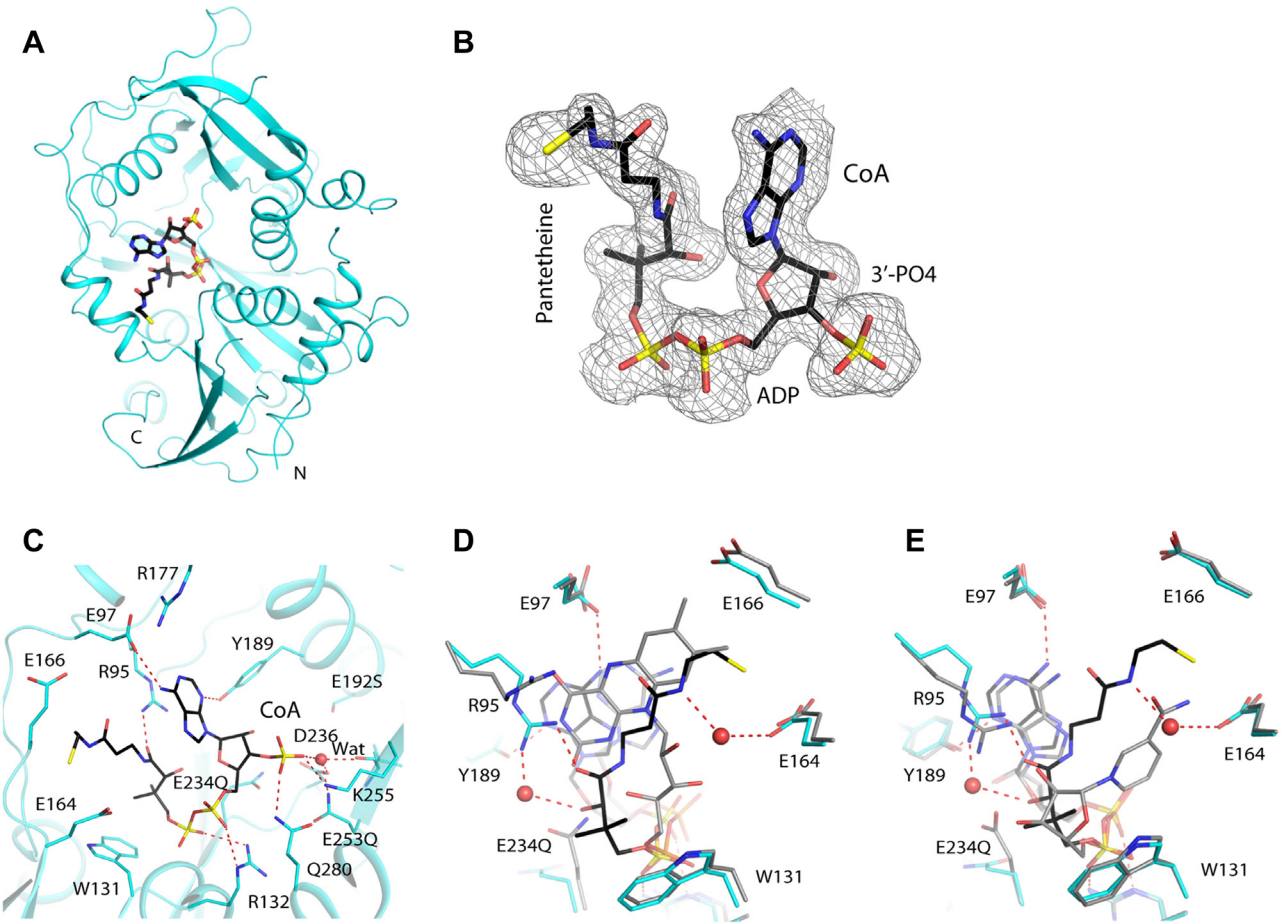
Crystal structures of mouse DXO in complex with CoA. (**A**) Overall structure of mouse DXO E192S/E234Q/E253Q mutant (cyan) in complex with CoA (in black for carbon atoms). (**B**) Simulated annealing omit *F*_o_– *F*_c_ electron density at 1.6 Å resolution for CoA, contoured at 2.5σ. (**C**) Detailed interactions between 3′-FADP and the active region of DXO. (**D**) Overlay of the active site region of mDXO in complex with CoA (in color) with that in complex with 3′-FADP (gray). (**E**) Overlay of the active site region of mDXO in complex with CoA (in color) with that in complex with 3′-NADP (gray). (gray, PDB: 5ULI).

To understand the molecular basis how DXO can recognize and remove 5′-end dpCoA caps, we determined the crystal structure of the E192S/E234Q/E253Q mutant of mDXO in complex with CoA at 1.6 Å resolution (Table 1). Like FADP and NADP, CoA is bound in the active site of DXO (Figure 4A), with good-quality electron density for the entire molecule (Figure 4B). It is also in a folded conformation, such that the amide bond in the pantetheine group is π-stacked against the adenine. The CoA molecule has extensive interactions with the active site of DXO (Figure 4C).

The overall structures of DXO in the CoA and FADP complexes are similar, with rms distance of 0.16 Å between their equivalent Cα atoms. The binding mode of the pyrophosphate and the 3′ phosphate group of CoA is similar to that of FADP, but the ribose and adenine have substantial differences in their orientation (Figure 4D). In fact, the binding mode of these two groups in CoA is similar to that in NADP where the oxygen atoms of pantothenate and ribose respectively are contacted by Arg95 (Figure 4E). The side chain of Tyr189 moves to maintain hydrogen-bonding to the N3 atom of adenine. The first half of the pantetheine group of CoA adopts a different path compared to the ribitol group of FADP, with the dimethyl moiety contacting Trp131. The second half of the pantetheine group follows the edge of the flavin ring that is closer to its ribitol group terminating with the β-mercaptoethylamine group being buried between the side chains of Glu164 and Glu166.

Comparisons among the CoA, FADP and NADP complexes show that the side chains of three residues undergo conformational changes to accommodate these different cap structures, while there are generally only small changes in the backbone conformations in the binding site. The largest difference is observed for Arg95. Its side chain assumes three different conformations in the three complexes, and its main chain also shows some changes. The conformations of its side chain in the CoA and NADP complexes clash with the flavin ring in the FADP complex. The second residue is E234(Q), which coordinates the second metal ion in the pU5 complex. In the NADP and FADP complexes, this side chain assumes a different rotamer and interacts with the cap structure. Finally, the side chain of Tyr189 undergoes conformational changes to establish a hydrogen bond to the N3 atom of the adenine.

### Detection of FAD-capped RNA in mammalian RNA by FAD-capQ

**Figure 5.**
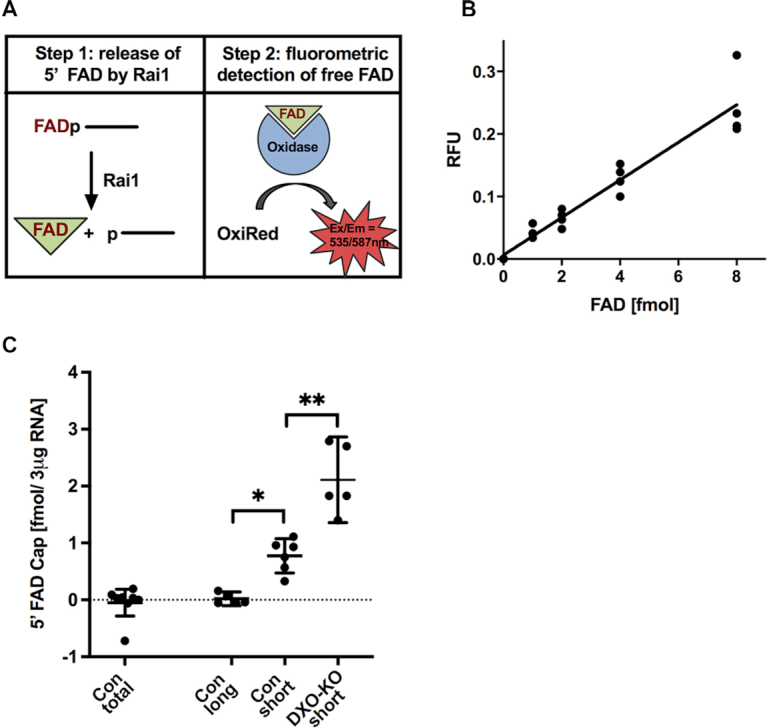
FAD-cap detection and quantitation assay. (**A**) Steps in FAD-capQ (see text). (**B**) FAD-capQ analysis of RNA generated *in vitro*. Increasing amounts of synthetic FAD-capped RNA were treated with SpRai1 and subject to FAD-capQ assay in the presence of 3 μg total RNA. Background signal detected without synthetic FAD-capped RNA was normalized to 1.0 and subtracted from each time point. (**C**) Total (50 μg), long (3 μg) and short (3 μg) RNAs from the indicated mammalian cells were subjected to FAD-capQ to detect levels of FAD caps. ‘Con’ indicates control knockout cells, ‘DXO-KO’ represents DXO knockout cells. Error bars represent ± SEM. Levels of statistical significance for at least five independent biological experiments were calculated using one-way Anova; *P = 0.018; **P = 0.0011.

We recently reported the development of NAD-capQ, a two-step enzymatic assay that determines NAD cap levels in cells by removal of the intact NAD cap from RNA, followed by quantitation of the NAD levels (18). We next tested whether an analogous methodology could be used to demonstrate the presence of FAD-capped RNAs in cells. We took advantage of the unique property of SpRai1 that removes the intact FAD without degrading the RNA body (Figure 1B) and coupled it with a fluorescent FAD detection system. We named this method FAD-cap detection and quantitation (FAD-capQ). FAD-capQ is a two-step procedure consisting of enzymatic treatment of RNA with SpRai1 to cleave the phosphodiester bond between the first and second encoded nucleotides within the RNA. This activity releases intact 5′-FAD that can be quantitated by a commercially available FAD fluorometric assay (BioVision, Inc.) (Figure 5A). The released FAD functions as an essential cofactor for an oxidase reaction and corresponding fluorescent emission of the OxiRed probe (Ex/Em = 535/587) within the assay (Figure 5A). The fluorometric assay parameters we utilized enabled detection of FAD in the fmol range, at levels approximately 10-fold lower than that reported by the manufacturer.

To validate FAD-capQ as a method for the detection of 5′-FAD caps, we utilized *in vitro* transcribed FAD-capped RNA. RNA at increasing concentrations was incubated with SpRai1 protein or a catalytically inactive SpRai1 with two amino acid substitutions, E199A and D201A (SpRai1 Mut) (20). The products of the reaction were subjected to the enzymatic assay and fluorescence in each sample at Ex/Em = 535/587 measured. The signal detected in samples treated with SpRai1 was above background levels (Figure 5B, Supplementary Figure S1, gray versus red bars) and significantly higher than fluorescence detected in samples treated with mutant SpRai1 (Supplementary Figure S1, red versus green bars), indicating that the observed values in the assay were derived from released FAD caps. Moreover, the feasibility of the FAD-capQ quantitative approach for FAD caps was further evident by the linear increase in fluorescence proportional to the amount of input FAD-capped RNA (Figure 5B, Supplementary Figure S1, red bars).

### Mammalian DXO is a deFADding enzyme in cells

To assess the level of FAD-capped RNA in mammalian cells, 50 μg of total RNA extracted from HEK293T cells was initially subjected to the FAD-capQ assay. However, no significant increase in fluorescence was detected above the background using the total RNA population as the starting material (Figure 5C). Since RNAs modified with nucleotide metabolites from bacterial cells are predominantly present in the short RNA fraction (11,12,30), we assessed whether FAD-caps can be detected on mammalian short RNAs. A LiCl precipitation approach coupled with silica-based RNA purification was used to separate RNA molecules into long (greater than ∼200 nts) and short RNA (less than ∼200 nts) populations (Supplementary Figure S2). This approach had an additional advantage of enriching the molar ratio of RNAs in the short RNA population relative to total RNA and potentially enhancing detection. Equal amounts of RNA isolated from each fraction were subjected to FAD-capQ. Intriguingly, the short RNA fraction from HEK293T cells revealed the presence of FAD caps at ∼1 fmol/3 μg of RNA (Figure 5C) while no significant fluorescence was detected above background with the long RNA fraction (Figure 5C) under the assay parameters employed. These findings demonstrate that FAD-capped RNAs are present in mammalian cells.

Based on the *in vitro* deFADding activity of mouse DXO (Figure 1A), we next tested whether DXO can function as a deFADding enzyme in cells. A pool of three monoclonal human HEK293T cell lines each harboring disruptions of the *DXO* gene in both alleles (DXO-KO) (15) was used. Interestingly, FAD-capQ analysis of short RNAs isolated from DXO-KO and control (Con) cells showed that total levels of FAD caps are ∼2-fold higher in cells lacking DXO (Figure 5C). This result supports a functional role for DXO in regulating the fate of FAD-capped RNAs in mammalian cells. Collectively, our data reveal the presence of FAD caps in mammalian cells and a role of DXO in removing these caps in cells.

## DISCUSSION

**Figure 6.**
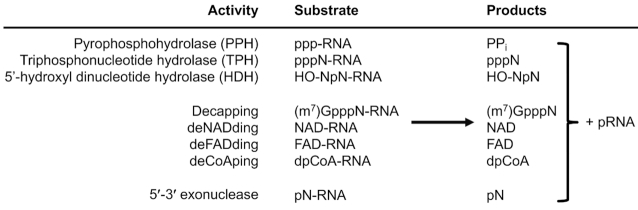
The diverse catalytic activity of DXO/Rai1 enzymes. The various catalytic activities of the DXO/Rai1 family enzymes are shown. They use the same catalytic machinery and share a common product, 5′-PO_4_ RNA (pRNA). References: pyrophosphohydrolase (PPH) (20); triphosphonucleotide hydrolase (TPH) (28); 5′-hydroxyl dinucleotide hydrolase (HDH) (41); decapping (26); deNADding (15); 5′-3′ exonuclease (27).

Non-canonical 5′-end caps are conserved RNA modifications found in bacteria and eukaryotes ranging from yeast to mammals and plants. The eukaryotic DXO/Rai1 decapping enzymes can eliminate diverse 5′ modifications from RNA, and here we show two additional activities for them: release of FAD and dpCoA caps from RNA (deFADding and deCoAping) (Figure 6). Despite the diverse substrate profile, all the decapping reactions produce one common product, 5′-monophosphate RNA (pRNA) and are carried out by a conserved core machinery, with two metal ions coordinating the scissile phosphate group and mediating the nucleophilic attack to initiate hydrolysis.

Besides the scissile phosphate, the structures of the various complexes determined so far suggest another phosphate group is recognized 5′ to the cleavage site, in the cap moiety itself, by the conserved Arg132 and Gln280 residues in DXO. This phosphate could be from the pyrophosphate in NAD-, FAD-, or dpCoA-capped RNA, the triphosphate of (m^7^)Gppp- or ppp-capped RNA, or the 5′ monophosphate of pRNA, which is the substrate for the 5′-3′ exonuclease activity. In addition, there is hydrophobic contact with the side chain of a bulky hydrophobic residue, Trp131 in DXO, among DXO/Rai1 family members. This includes the ribose of the nucleotide 5′ to the cleavage site, the ribose of the nicotinamide mononucleotide of NAD, the ribitol of FADP, and the dimethyl group of CoA.

The active site of DXO/Rai1 enzymes can be divided into two parts. The core catalytic machinery is at the bottom of a tunnel that extends to the surface of the enzymes. This tunnel is in contact with the body of the RNA substrate, recognizing only the backbone of single-stranded RNA but with no specificity in terms of its bases. The binding mode of the RNA body can show substantial differences among the enzymes (28). Similarly, the conformation of residues at the bottom of this tunnel that mediate the recognition of the cap structure can also undergo substantial changes. For example, residue Arg95 in DXO assumes different conformations to accommodate the different cap structures. While this residue is not conserved in Rai1 homologs and the loop containing this residue has structural differences in Rai1 enzymes compared to DXO, the loop does undergo conformational changes to accommodate and contact FAD and NAD in Rai1. Therefore, conformational rearrangements in this region may have an important role in recognizing the cap structure in DXO/Rai1 enzymes.

The structures of NAD, FAD and CoA are in a folded conformation when bound in the active site of DXO/Rai1. In comparison, the Nudix deNADding enzymes (NudC in bacteria and Nud12 in humans) bind the NAD in an extended conformation and cleaves between its pyrophosphate groups (14,19). It appears likely that the different binding modes of the NAD are linked to the different positions of the cleavage in these enzymes.

Despite sharing the core catalytic machinery and these conserved recognition mechanisms, DXO/Rai1 enzymes do not all possess the same range of substrates or cleavage activities (28). Variations of residues in the active site can dictate these activities, but it remains unclear how the different specificities for these enzymes are achieved.

In addition to NAD-capped RNAs (15,16), the recent demonstration of FAD caps on eukaryotic RNAs (32) expands our understanding of RNA epitranscriptomic regulatory networks. Using the CapQuant mass spectrometry-based approach with poly-adenylated RNA, Wang *et al.* demonstrated the presence of FAD caps on poly(A)+ mammalian RNAs in the fmol range (32). Our studies expand these findings to reveal FAD caps on short RNAs in human HEK293T cells at comparable fmol concentrations. Although the lack of detectable FAD caps on long RNAs in this study could be due to preferential deFADding of short RNAs by SpRai1, which we cannot rule out at present, it is more likely due to the smaller number of higher molecular weight RNA molecules relative to shorter RNAs at the same total weights used in the assay (3 μg), which may have made the longer RNAs below the detection limit of FAD-capQ. We were unable to detect FAD below 1 fmol levels, which itself was already an order of magnitude lower than that tested by the manufacturer and we could not reliably detect any lower levels. Collectively, our study and that of Wang *et al.* (32) reveal the presence of FAD-capped RNAs on both poly(A)+ and short RNAs in mammalian cells. At present, the nature of FAD-capped RNAs is not known. Identification of the specific RNAs harboring an FAD cap still awaits methodologies that can selectively isolate RNAs capped with FAD. Furthermore, in addition to the functional role of DXO in the modulation of NAD-capped RNAs (15), its impact on the levels of FAD-capped RNAs in cells implicates the DXO family of proteins as general regulators of non-canonical caps. Interestingly, despite the deCoAping activity of DXO *in vitro* (Figure 1A) dpCoA-capped RNAs were not detected in mRNAs extracted from unperturbed mammalian cells (32). Whether the lack of detection is indeed due to the absence of dpCoA-capped RNAs in mammalian cells or their susceptibility to DXO deCoAping activity that removes these caps in wild-type cells remains to be determined, although it may be speculated that the latter is more likely the case.

## Supplementary Material

gkaa297_Supplemental_File
