# Supplementary material for: DXO/Rai1 enzymes remove 5′-end FAD and dephospho-CoA caps on RNAs
Source: Nucleic Acids Res. 2020 May 6;48(11):6136–48. doi: 10.1093/nar/gkaa297 (PMC7293010; doi:10.1093/nar/gkaa297)
Supplement: gkaa297_Supplemental_File [file gkaa297_supplemental_file.pdf]

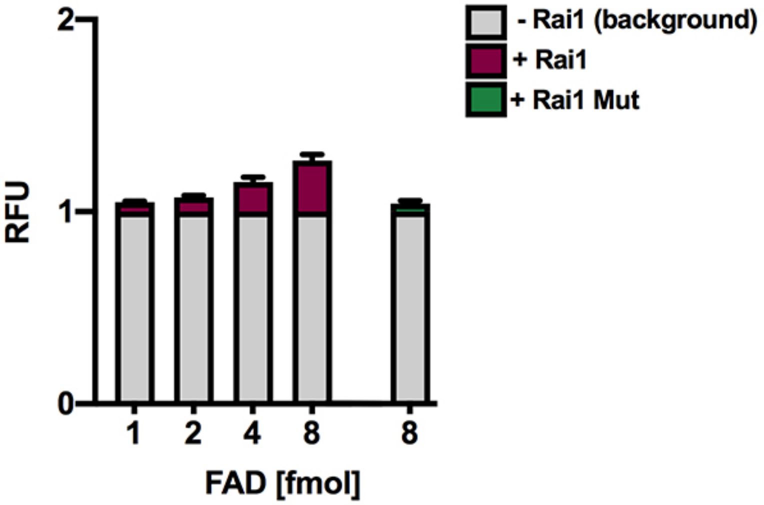

Fig. S1. FAD-capQ analysis of RNA generated *in vitro*. Synthetic FAD-capped RNA was treated with SpRai1 (red bar) or the catalytically inactive mutant of SpRai1 (green bar) and subject to FAD-capQ assay. Background signal detected without FAD-capped RNA, normalized to 1, is presented as grey bars.

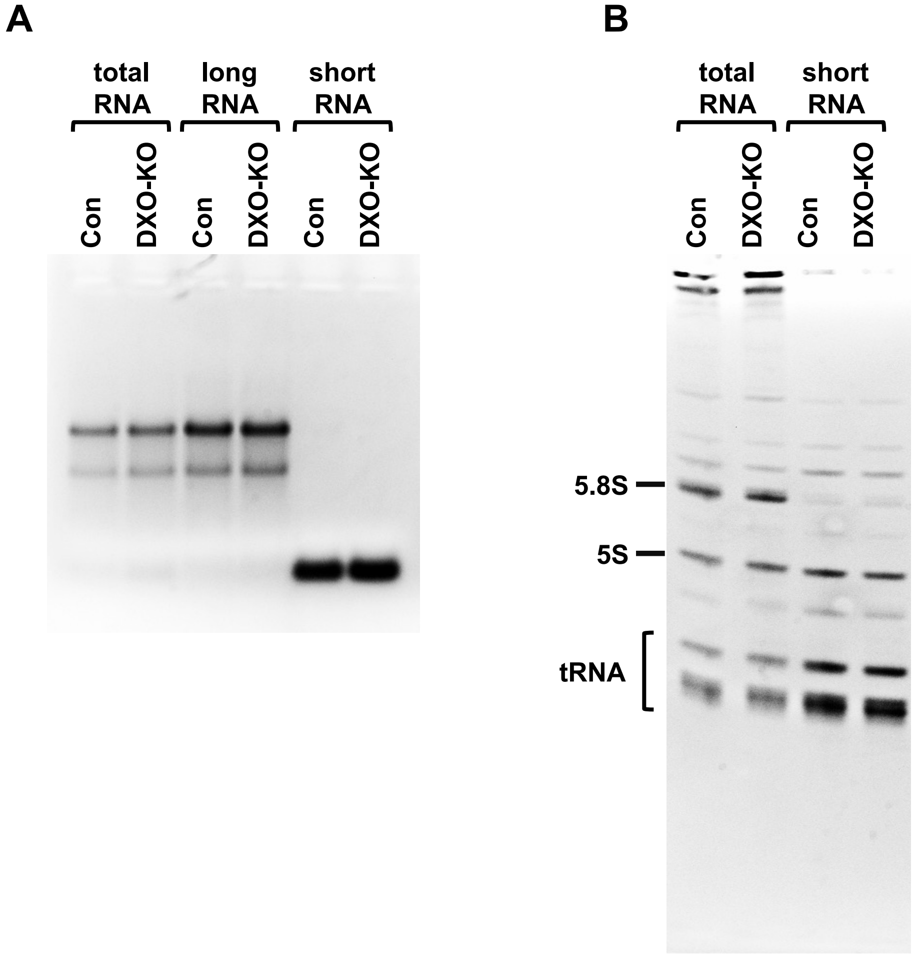

Fig. S2. RNA fractionation. Total, long and short RNA purified from HEK293T Con (Control) and DXO-KO cells were run on 1.5 % agarose gel (**A**) or 8 % denaturing polyacrylamide gel (**B**). 2 μg of RNA from each fraction was run on the gel in (**A**) and 10 μg total RNA and 2 μg short RNA was run in (**B**).
